# Seroprevalence and Factors Associated with *Toxoplasma gondii* Seropositivity in Small Ruminants in Northeastern Portugal

**DOI:** 10.3390/ani16172691

**Published:** 2026-08-28

**Authors:** Tifany Pereira, Carina Rodrigues, Maria João Caldeira, Filipa Teixeira Rodrigues, João Jacob-Ferreira, Ana Patrícia Lopes, Hélder Quintas

**Affiliations:** 1 Associate Laboratory for Sustainability and Technology in Mountains (SusTEC), Mountain Research Centre (CIMO), Polytechnic Institute of Bragança (IPB), Campus de Santa Apolónia, 5300-253 Bragança, Portugal; tifanyapereira@ipb.pt (T.P.); up202412059@edu.icbas.up.pt (M.J.C.); filipatr@ipb.pt (F.T.R.); joao.ferreira.vet@gmail.com (J.J.-F.); 2 Department of Veterinary Sciences, School of Agrarian and Veterinary Sciences (ECAV), University of Trás-os-Montes and Alto Douro (UTAD), 5000-801 Vila Real, Portugal; aplopes@utad.pt; 3 LiveWell, Research Centre for Active Living and Wellbeing, School of Health, Polytechnic Institute of Bragança (IPB), Campus de Santa Apolónia, 5300-253 Bragança, Portugal; carina@ipb.pt; 4 Abel Salazar Biomedical Sciences Institute (ICBAS), University of Porto, Rua de Jorge Viterbo Ferreira 228, 4050-313 Porto, Portugal; 5 Associate Laboratory for Animal and Veterinary Sciences (AL4AnimalS), Animal and Veterinary Research Centre (CECAV), Department of Veterinary Sciences, School of Agrarian and Veterinary Sciences (ECAV), University of Trás-os-Montes and Alto Douro (UTAD), 5000-801 Vila Real, Portugal

**Keywords:** *Toxoplasma gondii*, seroprevalence, sheep, goats, associated factors, ELISA, northeastern Portugal

## Abstract

*Toxoplasma gondii* infection is relevant to animal production, food safety, and public health, but current epidemiological data for small ruminants in northeastern Portugal are limited. This cross-sectional study estimated seroprevalence and investigated factors associated with seropositivity in 2678 animals from 149 flocks sampled between May 2022 and December 2024. Serum samples were tested using an indirect enzyme-linked immunosorbent assay, and associated factors were evaluated using mixed-effects logistic regression with flock as a random effect. The overall true seroprevalence was estimated at 21.5% (apparent seroprevalence: 21.6%). At the flock level, 83.9% (125/149) of the sampled flocks were seropositive. True seroprevalence was higher in sheep than in goats (23.4% versus 14.4%), and seropositivity was detected in all surveyed municipalities. In the multivariable model, higher odds of seropositivity were observed in sheep (adjusted odds ratio 1.74), adult animals (2.78), non-native breeds (1.84), animals not kept in permanent housing (3.16), and animals from farms with cats (1.69). These findings indicate exposure throughout the surveyed area and associations with both host-related and farm-level characteristics. Strengthening farm biosecurity, limiting contamination of feed and water, and integrating animal health, environmental, and food safety measures may contribute to prevention within a One Health framework.

## Simple Summary

*Toxoplasma gondii* is a parasite that can infect sheep and goats and is relevant to animal production and food safety. Information on its occurrence in sheep and goat farms in northeastern Portugal is limited. This study examined blood samples from 2678 animals belonging to 149 flocks and collected information on animal characteristics and farm practices. Antibodies indicating prior exposure to the parasite were detected in 21.6% of the animals, and flocks containing at least one antibody-positive animal were identified in every municipality studied. Sheep, adult animals, non-native breeds, animals not kept permanently indoors, and animals from farms where cats were present had significantly higher odds of seropositivity. Other management- and farmer-related characteristics were not significantly associated with seropositivity after adjustment for the remaining variables. These findings indicate that *T. gondii* exposure is widespread throughout the study area and that seropositivity is associated with both animal- and farm-level characteristics. The findings provide evidence to support preventive measures, including protecting feed and water from contamination, limiting cat access to livestock and feed-storage areas, and improving farm hygiene and awareness, thereby benefiting animal health, environmental protection, and food safety.

## 1. Introduction

Toxoplasmosis is a globally distributed zoonosis caused by the obligate intracellular protozoan parasite *Toxoplasma gondii*, which has a heteroxenous life cycle involving felids as definitive hosts and a broad range of warm-blooded animals, including humans, sheep, and goats, as intermediate hosts [1]. Sexual reproduction occurs in the intestinal epithelium of felids, resulting in the shedding of unsporulated oocysts in their faeces [2,3]. After sporulation, oocysts become infective and may persist in the environment, contaminating soil, water, pasture, and feed [4]. Following ingestion by an intermediate host, the parasite initially replicates as tachyzoites, which disseminate throughout the host and subsequently differentiate into bradyzoites contained within persistent tissue cysts [2,5]. Although infection is generally subclinical in immunocompetent hosts, severe disease may occur in immunocompromised individuals. Additionally, infection during gestation may result in transplacental transmission and adverse reproductive outcomes [1,5].

In sheep and goats, *T. gondii* infection is of particular veterinary importance because vertical transmission during gestation can lead to placental and foetal infection, resulting in embryonic death, mummification, abortion, stillbirth, or the birth of weak offspring [6]. These reproductive consequences can reduce flock productivity and cause substantial economic losses, particularly in production systems dependent on lamb and kid production [7,8,9]. Small ruminants are also relevant from a public health perspective, as humans may be exposed through the consumption of raw or undercooked meat and unpasteurized milk from infected animals [10,11]. Given its implications for animal health, public health, and environmental transmission, toxoplasmosis represents a relevant One Health concern [1,12].

Exposure to *T. gondii* in small ruminants is multifactorial and may be influenced by the interplay between animal-related characteristics, environmental conditions, and farm-management practices [7,13]. Animal-level factors, including species, age, sex, and breed, have been investigated as potentially associated with seropositivity [14,15,16]. At the farm level, grazing and outdoor access, housing conditions, and the presence of cats may influence exposure to the parasite, particularly through the contamination of pasture, feed, and water with oocysts [7,13,14,16]. As herbivorous intermediate hosts, sheep and goats are mainly exposed to *T. gondii* through the ingestion of oocysts contaminating pasture, feed, or water, although vertical transmission may also occur [7]. Practices concerning reproductive areas, feed and water hygiene, and the management, cleaning, and disinfection of facilities and equipment are also relevant components of farm biosecurity [17]. Farmer education and access to training and veterinary support may influence the implementation of biosecurity measures [17,18]. More broadly, lower educational attainment has been associated with higher *T. gondii* seropositivity among blood donors in Portugal [19].

In Portugal, sheep and goat farming is a traditional livestock activity of considerable economic importance, mainly oriented towards meat, milk, and wool production [20]. In northern Portugal, small-ruminant farming is frequently associated with small family-run farms, relatively small flock sizes, the use of natural pastures, and the presence of native breeds. Extensive and semi-extensive production systems predominate, with the latter generally combining daytime grazing with overnight housing as an adaptation to the region’s climatic and topographic conditions [21,22,23]. This production context is particularly relevant in Trás-os-Montes, northeastern Portugal, where regular outdoor access may favour exposure to *T. gondii* oocysts present in contaminated soil, pastures, feed, and water [7].

Previous serological studies conducted in northern and northeastern Portugal detected anti-*T. gondii* antibodies in livestock, companion animals, wildlife, and women of childbearing age, documenting exposure across different host populations in the region [24,25,26,27,28]. Among small ruminants, Sousa et al. [29] reported a seroprevalence of 17.1% in sheep from the municipality of Vinhais. In a separate study of animals intended for human consumption from northern Portugal, Lopes et al. [28] found seroprevalences of 33.6% in sheep and 18.5% in goats. Despite these contributions, up-to-date regional epidemiological evidence jointly assessing seroprevalence and factors associated with seropositivity in sheep and goats from northeastern Portugal remains limited.

Against this background, the present study aimed to estimate the seroprevalence of *T. gondii* in sheep and goats from northeastern Portugal and to identify animal-, farm-, management-, and farmer-related factors associated with seropositivity. By providing an updated epidemiological characterisation of *T. gondii* seropositivity in small ruminants from this region, this study may contribute to the development of more targeted prevention and surveillance strategies within a One Health framework.

## 2. Materials and Methods

### 2.1. Study Area, Study Design and Sampling

A cross-sectional study was conducted to estimate *T. gondii* seroprevalence and identify factors associated with seropositivity in small ruminants from the Trás-os-Montes region, northeastern Portugal. Sampling was carried out between May 2022 and December 2024.

A two-stage cluster sampling design was employed, in which flocks served as primary sampling units and individual animals as secondary sampling units. Small-ruminant flocks were selected from the official Portuguese animal health database, PISA.net (Digidelta Software, Leiria, Portugal). To ensure geographical representativeness, flocks were randomly selected in proportion to the distribution of holdings across the municipalities included in the study area. Only flocks comprising at least 20 animals were eligible for inclusion.

The minimum sample size required for prevalence estimation was calculated using the formula *n* = *Z*^2^*P*(1 − *P)*/*d*^2^, where *n* represents the required sample size, *Z* corresponds to the value associated with a 95% confidence level, *P* is the expected prevalence, and *d* is the desired absolute precision [30]. An expected prevalence of 15% was assumed based on previous epidemiological studies conducted in Portuguese small-ruminant populations [31]. Although this calculation assumes simple random sampling, it was used as a practical approximation for study planning. The clustered structure of the data was subsequently accounted for during the analytical phase using mixed-effects models that incorporated flock as a random effect. Because no reliable estimate of the design effect was available for this population during the planning stage, no inflation factor was applied to the initial sample size calculation. The final sample size exceeded the minimum number of animals required for the study.

A total of 2678 sheep and goats from 149 flocks were included in the study, comprising 2111 sheep and 567 goats. Because complete flock sampling was not feasible, a random within-flock sampling strategy was implemented. Within each flock, animals were selected by simple random sampling using random numbers generated from complete flock inventories. Only animals aged six months or older were eligible for sampling. This sampling strategy preserved the hierarchical structure of the dataset, with individual animals nested within flocks.

Sampling was integrated into routine flock health visits, conducted by the veterinarians responsible for each flock within the framework of the Portuguese national animal health surveillance, control, and eradication programmes. No additional handling or restraint procedures were performed exclusively for the purposes of this study.

### 2.2. Epidemiological Data Collection

Potential factors associated with *T. gondii* seropositivity were investigated using a structured epidemiological questionnaire administered during flock visits. Before questionnaire administration and biological sample collection, flock owners were informed about the study objectives, procedures, voluntary participation, and data confidentiality, and written informed consent was obtained. The questionnaire consisted of closed-ended questions designed to collect information on flock characteristics, including species composition, production system, management practices, housing conditions, feeding and watering systems, reproductive management, biosecurity measures, the presence of domestic and wild animals of potential epidemiological relevance, and farmer-related characteristics.

Questionnaire responses were recorded for each flock and subsequently linked to the corresponding serological results using a unique flock identification code.

### 2.3. Serological Analysis

Blood samples (10 mL) were collected from the jugular vein of each animal using vacuum tubes containing a clot activator (Vacutainer^®^, Becton Dickinson, Plymouth, UK). Samples were allowed to clot at room temperature before centrifugation, and serum was separated and stored at −20 °C until analysis.

Serological testing was performed to detect IgG antibodies against *T. gondii* using a commercial indirect enzyme-linked immunosorbent assay (ELISA), ID Screen^®^ Toxoplasmosis Indirect Multi-species (IDvet, Grabels, France), following the manufacturer’s instructions. This assay is designed to detect anti-*T. gondii* antibodies in multiple animal species and has been extensively used in epidemiological studies of toxoplasmosis in livestock. According to published validation data, the assay demonstrated diagnostic sensitivity of 98.36% and diagnostic specificity of 99.42% compared with the modified agglutination test (MAT) [32].

Results were interpreted according to the manufacturer’s instructions. Samples with S/P% values < 40% were considered negative, samples with S/P% values between 40% and 50% were considered doubtful, and samples with S/P% values ≥ 50% were considered positive.

### 2.4. Statistical Analysis

Data were entered into Microsoft Excel^®^ for Microsoft 365 (Microsoft Corporation, Redmond, WA, USA) and checked for completeness and consistency prior to analysis. Serological results were merged with the epidemiological questionnaire database to create the final dataset.

Apparent seroprevalence was estimated at both the animal and flock levels. Animal-level apparent seroprevalence was defined as the proportion of seropositive animals, whereas flock-level seroprevalence was defined as the proportion of sampled flocks with at least one seropositive animal. For animal-level apparent seroprevalence, 95% confidence intervals (95% CIs) were estimated using a logit-based approach in the R survey package, with flock specified as the clustering unit to account for the non-independence of animals sampled within the same flock. True seroprevalence at the animal level and its corresponding 95% CI were estimated using the Rogan–Gladen correction, based on the published diagnostic sensitivity and specificity of the ELISA [32] and applied to the apparent seroprevalence estimate and to the lower and upper limits of its cluster-adjusted 95% CI. At the flock level, apparent seroprevalence was estimated overall and according to flock composition (sheep, goat, or mixed sheep–goat flocks), with exact binomial 95% confidence intervals. True seroprevalence was not estimated at the flock level because the available diagnostic sensitivity and specificity estimates refer to individual-animal testing.

Descriptive statistics were used to summarise the distribution of *T. gondii* seropositivity among animals and flocks. Age was categorised into two groups for statistical analysis: young (6–18 months) and adult (>18 months). Associations between serological status and categorical explanatory variables were initially explored using Pearson’s chi-square (χ^2^) test. These analyses were considered exploratory and did not account for clustering.

Given the hierarchical structure of the dataset, with animals clustered within flocks, analysis of factors associated with seropositivity was performed at the individual-animal level using mixed-effects logistic regression models. Individual serological status was treated as the binary outcome variable, and flock was included as a random intercept to account for intra-flock correlation. The intraclass correlation coefficient (ICC) was calculated on the latent scale as σ^2^flock/(σ^2^flock + π^2^/3), where σ^2^flock represents the variance in the flock-level random intercept.

For analytical purposes, explanatory variables were grouped into animal-, farm-, management-, and farmer-related domains. Biological relevance was assessed a priori based on published evidence and included host-related characteristics, plausible routes of environmental exposure to *T. gondii* oocysts, and farm biosecurity practices. Variables with a *p*-value < 0.20 in the exploratory analyses, together with those considered biologically relevant, were entered into univariable mixed-effects logistic regression analyses. Variables selected from the univariable mixed-effects analyses were subsequently included in the multivariable mixed-effects logistic regression model. Model building followed a backward elimination procedure, whereby variables were sequentially removed based on statistical significance and their impact on model stability.

Potential confounding was evaluated by examining changes in the estimated odds ratios (ORs) of the remaining variables. Variables that resulted in a change of ≥10% in at least one OR estimate were retained in the final model, irrespective of their statistical significance. As additional sensitivity analyses, sampling year and sampling season were separately added to the final multivariable mixed-effects logistic regression model to assess whether temporal heterogeneity during the study period materially affected the estimated associations. Municipality-specific seroprevalence was examined descriptively, given the unequal number of sampled flocks across municipalities. The potential species × municipality interaction was considered but was not included in the multivariable model because several species–municipality combinations were absent or sparsely represented. Model fit was assessed using likelihood ratio tests and by evaluating the flock-level random-effect variance. Statistical significance was defined as *p* < 0.05 throughout the analyses.

Statistical analyses were performed using JMP^®^ Statistical Discovery software, version 19 (SAS Institute Inc., Cary, NC, USA), and R version 4.6.1 (R Foundation for Statistical Computing, Vienna, Austria) with the survey package to estimate cluster-adjusted 95% confidence intervals for animal-level apparent seroprevalence, with flock specified as the clustering unit. The spatial distribution of apparent *T. gondii* seroprevalence by municipality and the geographical location of sampled flocks were mapped using QGIS software, version 4.0.3 (Norrköping; QGIS Development Team).

## 3. Results

### 3.1. Seroprevalence of Toxoplasma gondii

No serum samples yielded doubtful ELISA results. The overall apparent seroprevalence of *T. gondii* in small ruminants from northeastern Portugal was 21.6% (579/2678; 95% CI: 18.7–24.8). Apparent seroprevalence was higher in sheep than in goats, reaching 23.5% (496/2111; 95% CI: 20.2–27.2) and 14.6% (83/567; 95% CI: 9.7–21.5), respectively. After adjustment using the published diagnostic sensitivity and specificity values of the ELISA, the estimated true seroprevalence was 21.5% (95% CI: 18.5–24.8) overall, corresponding to 23.4% (95% CI: 20.0–27.2) in sheep and 14.4% (95% CI: 9.3–21.4) in goats.

At the flock level, 125 of the 149 sampled flocks were seropositive, corresponding to an apparent seroprevalence of 83.9% (95% CI: 77.0–89.4). Of the 149 flocks, 112 were classified as sheep flocks, 31 as goat flocks, and 6 as mixed sheep–goat flocks. Seropositivity was detected in 98 sheep flocks (87.5%; 95% CI: 79.9–93.0), 21 goat flocks (67.7%; 95% CI: 48.6–83.3), and all 6 mixed flocks (100.0%; 95% CI: 54.1–100.0) (Table 1).

### 3.2. Spatial Distribution of Toxoplasma gondii Seroprevalence

**Figure 1:**
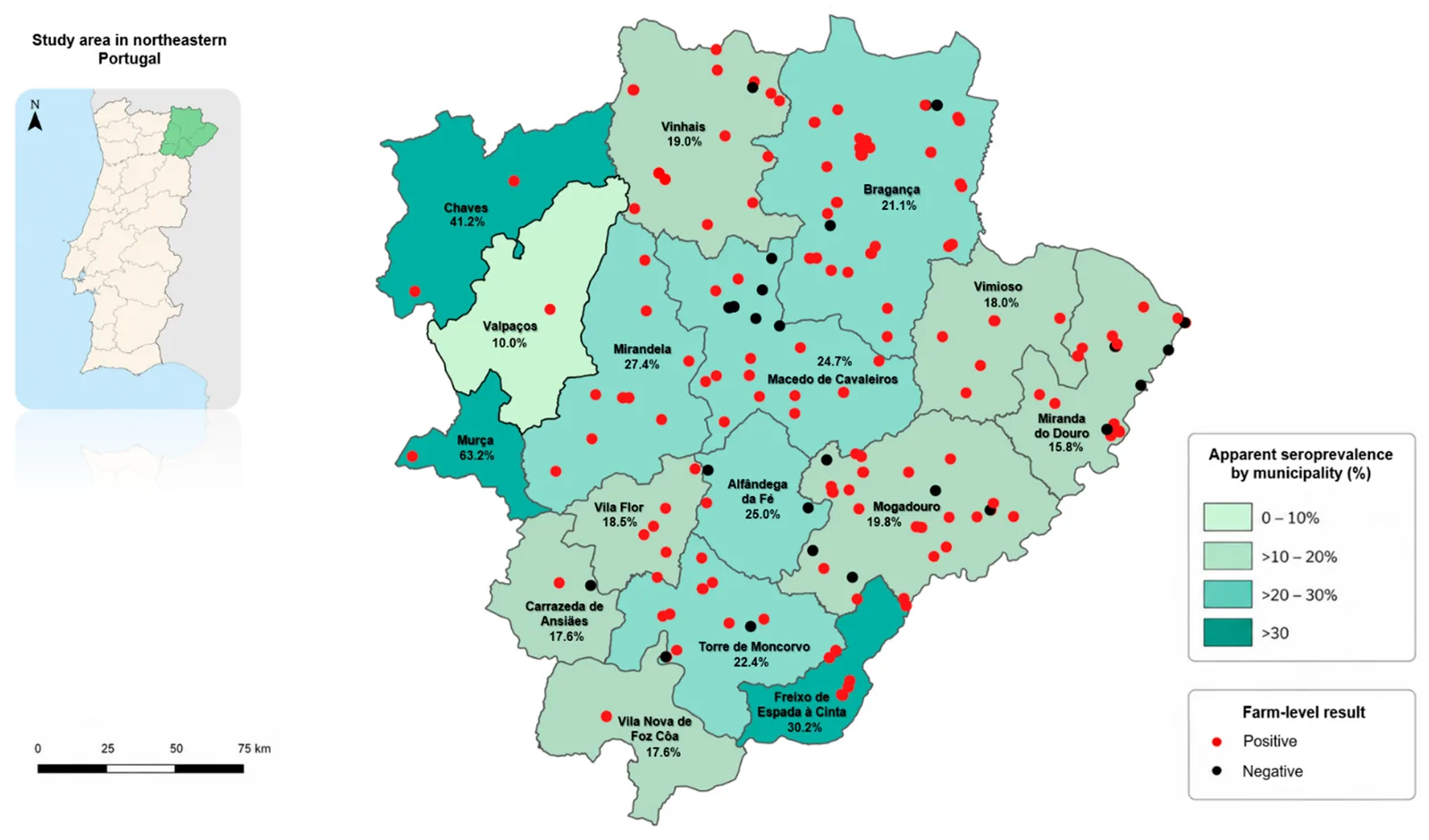
Spatial distribution of the apparent *T. gondii* seroprevalence by municipality in northeastern Portugal. Municipalities are coloured according to the apparent seroprevalence estimated from the sampled animals in each municipality. Red and black dots represent the geographical location of seropositive and seronegative flocks, respectively.

The spatial distribution of the apparent *T. gondii* seroprevalence by municipality is presented in Figure 1. Most municipalities showed apparent seroprevalence rates of approximately 15% to 30%, although they ranged from 10.0% in Valpaços to 63.2% in Murça. The geographical distribution of the 149 sampled flocks and their serological status is also presented, showing that *T. gondii*-positive flocks were detected throughout the study area. Because the number of sampled flocks varied among municipalities, municipality-specific seroprevalence estimates were interpreted descriptively and were not used to infer differences in risk between municipalities.

### 3.3. Exploratory Analysis of Factors Associated with Toxoplasma gondii Seropositivity

Exploratory analysis identified several factors significantly associated with *T. gondii* seropositivity (Table 2). At the animal level, seroprevalence was significantly higher in sheep than in goats (23.5% vs. 14.6%; *p* < 0.001), in adult animals than in young animals (26.5% vs. 13.0%; *p* < 0.001), and in non-native breeds than in native breeds (25.7% vs. 17.0%; *p* < 0.001).

At the farm level, permanent housing was reported in 7 of the 149 sampled flocks (4.7%). Animals raised without permanent housing showed significantly higher seroprevalence than those raised under permanent housing (22.2% vs. 7.9%; *p* < 0.001). Likewise, cats were present for 61 flocks (40.9%), and the presence of cats on the farm was associated with higher seroprevalence (25.4% vs. 19.0%; *p* < 0.001).

Regarding management practices, a dedicated maternity area was available in 46 flocks (30.9%), and the absence of a dedicated maternity area was associated with higher seroprevalence (22.8% vs. 19.1%; *p* = 0.033). Sharing water troughs with other animals was reported in 61 flocks (40.9%) and the use of equipment from other farms in 59 flocks (39.6%); both practices were significantly associated with *T. gondii* seropositivity (*p* = 0.025 and *p* = 0.022, respectively).

Farmer-related characteristics were also associated with seropositivity. Livestock production training was reported for the owners of 25 flocks (16.8%), and farms whose owners had received livestock production training showed lower seroprevalence than those without such training (16.8% vs. 22.6%; *p* = 0.007). Likewise, owners of 24 flocks (16.1%) had completed secondary or higher education, and farms whose owners had completed secondary or higher education showed lower seroprevalence than those whose owners had lower educational attainment (18.1% vs. 22.3%; *p* = 0.046).

No significant associations were observed between *T. gondii* seropositivity and sex, production purpose, production system, modern or technified facilities, contact with wildlife species, introduction of breeding males from other farms, farm hygiene, water trough hygiene, or regular veterinary assistance (all *p* > 0.05).

### 3.4. Factors Associated with Toxoplasma gondii Seropositivity

The results of the univariable and multivariable mixed-effects logistic regression analyses are presented in Table 3. After adjustment for flock-level clustering, five variables remained independently associated with *T. gondii* seropositivity. Sheep had higher odds of seropositivity than goats (aOR = 1.74; 95% CI: 1.08–2.79; *p* = 0.023). Adult animals had higher odds of seropositivity than young animals (aOR = 2.78; 95% CI: 2.20–3.51; *p* < 0.001). Likewise, non-native breeds had higher odds of seropositivity than native breeds (aOR = 1.84; 95% CI: 1.24–2.72; *p* = 0.003). Animals raised on farms without permanent housing had higher odds of seropositivity than those raised on farms with permanent housing (aOR = 3.16; 95% CI: 1.04–9.60; *p* = 0.042). The presence of cats on the farm was also associated with higher odds of seropositivity (aOR = 1.69; 95% CI: 1.11–2.57; *p* = 0.015). The estimated variance of the flock-level random intercept was 0.948, corresponding to an ICC of 0.224 (22.4%), indicating appreciable clustering of seropositivity within flocks. In additional sensitivity analyses, sampling year and sampling season were not significantly associated with *T. gondii* seropositivity when separately added to the final multivariable model (*p* = 0.2925 and *p* = 0.7890, respectively). Adjustment for sampling year did not materially affect the estimated associations. Adjustment for sampling season also produced little change in most effect estimates, although the estimate for permanent housing was attenuated, warranting cautious interpretation of this association.

## 4. Discussion

In the present study, anti-*T. gondii* antibodies were detected in 21.6% of the sampled small ruminants, and seropositive flocks were identified in all surveyed municipalities. These findings document exposure to *T. gondii* throughout the study area and provide updated information on the epidemiological situation in small-ruminant production systems in northeastern Portugal.

Among sheep, the apparent seroprevalence in the present study (23.5%) was higher than the 17.1% reported by Sousa et al. [29], but lower than the 33.6% reported by Lopes et al. [28] in animals from northern Portugal intended for human consumption. In goats, the apparent seroprevalence in the present study (14.6%) was also lower than the 18.5% reported by Lopes et al. [28]. Almeida et al. [31] reported higher seroprevalence estimates in a longitudinal cohort of Serra da Estrela sheep from central Portugal, increasing from 57.7% in 2015 to 69.1% in 2016. These differences should be interpreted cautiously, as previous Portuguese studies differed in their sampled populations, study designs, and serological approaches, including the use of MAT or ELISA.

Several epidemiological studies of small ruminants have used logistic regression or mixed-effects modelling approaches to evaluate host- and farm-level factors associated with *T. gondii* seropositivity [14,15,16,33,34,35,36].

Among the host-related factors associated with seropositivity in the multivariable model, sheep had higher odds of seropositivity than goats, consistent with findings from northern Greece, Switzerland, northern Portugal, and Pakistan [15,28,33,37]. This difference may partly reflect feeding behaviour, as sheep graze closer to the ground and may consequently have greater contact with oocysts contaminating pasture or soil, whereas goats tend to browse higher vegetation [15,38]. However, the association between species and seropositivity has not been consistent across studies [14].

Age was also associated with seropositivity, with adult animals showing higher odds than young animals, a pattern also reported in northern Portugal [28] and identified in multivariable analyses of other small-ruminant populations [15,33,34]. This association is consistent with cumulative exposure, as older animals have had more opportunities to ingest oocysts from contaminated pasture, soil, feed, or water throughout their lifetime. The age-related pattern observed in the present study therefore reinforces the importance of environmental exposure in the transmission of *T. gondii* among small ruminants.

Breed origin was another host-related factor associated with seropositivity, with non-native breeds showing higher odds than native breeds. Associations between breed and *T. gondii* seropositivity have also been reported in Romania, Estonia, and South Africa, although the findings were not consistent across the populations studied [14,34,35]. In the present study, this association may reflect unmeasured differences in management or environmental exposure, and an intrinsic breed effect cannot be inferred.

Among the farm-level factors associated with seropositivity in the multivariable model, animals not kept in permanent housing had higher odds of seropositivity than those kept in permanent housing. However, this finding should be interpreted with caution given the limited representation of the permanent-housing category and the attenuation of the association observed after adjustment for sampling season in the sensitivity analysis. Production system was identified as a factor associated with seropositivity in northern Italy, with lower seropositivity observed in intensive than in extensive or semi-intensive systems [16]. In Algerian sheep, semi-extensive management was associated with higher odds of seropositivity in logistic regression analysis [36]. These findings are biologically plausible because, in herbivorous intermediate hosts such as sheep and goats, horizontal exposure occurs primarily through the ingestion of oocysts present in the environment, making contamination of pasture, soil, feed, and water particularly relevant; vertical transmission may also occur [7]. Nevertheless, permanent housing does not completely prevent exposure, as feed and water may still become contaminated, and cats may enter livestock facilities [7].

The association with cat presence is also epidemiologically plausible, as felids are the definitive hosts of *T. gondii* and may contaminate livestock facilities, feed storage areas, water sources, or pastures with oocysts. Similar findings have been reported in sheep and goats from Romania, in indoor-housed dairy goats in the Netherlands, and in sheep from Algeria [14,36,39]. Nevertheless, the reported presence of cats does not establish that these animals were infected or shedding oocysts and should therefore be interpreted as an indicator of potential environmental exposure.

Beyond the factors identified in the multivariable model, several management- and farmer-related characteristics represented potential routes of environmental contamination or factors influencing the implementation of preventive practices. The absence of a dedicated maternity area, sharing of water troughs, use of equipment from other farms, and lack of livestock-production training were associated with seropositivity in the exploratory analysis, but were not independently associated in the adjusted model. No clear associations were observed for sex, production purpose, wildlife contact, farm or water-trough hygiene, or regular veterinary assistance. These findings do not exclude the general relevance of such practices to farm biosecurity but indicate that their specific contribution to *T. gondii* seropositivity was not demonstrated in the population studied.

From a practical perspective, the findings support the use of measures to limit contamination of feed, water, and livestock environments with *T. gondii* oocysts. Particular attention should be given to restricting cat access to feed storage and animal housing areas while maintaining appropriate farm hygiene and biosecurity practices. Further studies integrating animal, environmental, and farm-management data would help clarify the pathways of exposure operating in regional production systems.

### Study Limitations

This study has some limitations that should be considered when interpreting the findings. Owing to its cross-sectional design, the observed associations cannot establish temporal or causal relationships. In addition, the limited representation of some exposure categories may have affected the precision of the corresponding estimates. Finally, the unequal distribution of sampled flocks across municipalities limits direct comparisons between municipality-specific seroprevalence estimates.

## 5. Conclusions

This study provides updated evidence of *T. gondii* exposure in small ruminants in northeastern Portugal, with an overall true seroprevalence estimated at 21.5%, with 83.9% of the sampled flocks being seropositive. In the multivariable model, higher odds of seropositivity were observed in sheep, adult animals, non-native breeds, animals not kept in permanent housing, and animals from farms where cats were present, although the association with permanent housing should be interpreted cautiously. These findings highlight the contribution of both host-related and farm-level factors and support preventive measures aimed at reducing potential environmental contamination of feed, water, and livestock areas within a One Health framework.

## Figures and Tables

**Table 1:** Animal- and flock-level seroprevalence of *Toxoplasma gondii* in small ruminants from northeastern Portugal.

	Positive/Total (*n*/*N*)	Apparent Seroprevalence % (95% CI)	True Seroprevalence % (95% CI)
A. Animal level			
Sheep	496/2111	23.5 (20.2–27.2)	23.4 (20.0–27.2)
Goats	83/567	14.6 (9.7–21.5)	14.4 (9.3–21.4)
Total	579/2678	21.6 (18.7–24.8)	21.5 (18.5–24.8)
B. Flock level			
Sheep flocks	98/112	87.5 (79.9–93.0)	—
Goat flocks	21/31	67.7 (48.6–83.3)	—
Mixed flocks	6/6	100.0 (54.1–100.0)	—
Total	125/149	83.9 (77.0–89.4)	—

—, not applicable.

**Table 2:** Exploratory analysis of factors associated with *Toxoplasma gondii* seropositivity in small ruminants from northeastern Portugal.

Variables	Flocks, *n* (%)	Animals, *n* (%)	Seropositive Animals *n* (%)	*p*-Value
Animal				
Species				**<0.001**
Sheep	—	2111 (78.8)	496 (23.5)	
Goats	—	567 (21.2)	83 (14.6)	
Sex				0.650
Female	—	2529 (94.4)	549 (21.7)	
Male	—	149 (5.6)	30 (20.1)	
Animal age				**<0.001**
Young	—	965 (36.0)	125 (13.0)	
Adult	—	1713 (64.0)	454 (26.5)	
Production purpose				0.545
Meat	—	1743 (65.1)	383 (22.0)	
Dairy	—	935 (34.9)	196 (21.0)	
Native breed				**<0.001**
Yes	—	1251 (46.7)	212 (17.0)	
No	—	1427 (53.3)	367 (25.7)	
Farm				
Production system				0.198
Intensive	3 (2.0)	55 (2.1)	8 (14.5)	
Semi-extensive	146 (98.0)	2623 (97.9)	571 (21.8)	
Permanent housing				**<0.001**
Yes	7 (4.7)	114 (4.3)	9 (7.9)	
No	142 (95.3)	2564 (95.7)	570 (22.2)	
Modern/technified facilities				0.499
Yes	65 (43.6)	1143 (42.7)	240 (21.0)	
No	84 (56.4)	1535 (57.3)	339 (22.1)	
Presence of cats on the farm				**<0.001**
Yes	61 (40.9)	1102 (41.2)	280 (25.4)	
No	88 (59.1)	1576 (58.9)	299 (19.0)	
Contact with wildlife species				0.293
Yes	103 (69.1)	1879 (70.2)	396 (21.1)	
No	46 (30.9)	799 (29.8)	183 (22.9)	
Introduction of breeding males from other farms				0.835
Yes	11 (7.4)	209 (7.8)	44 (21.1)	
No	138 (92.6)	2469 (92.2)	535 (21.7)	
Management practices				
Dedicated maternity area				**0.033**
Yes	46 (30.9)	842 (31.4)	161 (19.1)	
No	103 (69.1)	1836 (68.6)	418 (22.8)	
Farm hygiene				**0.496**
Adequate	62 (41.6)	1114 (41.6)	248 (22.3)	
Needs improvement	87 (58.4)	1564 (58.4)	331 (21.2)	
Water trough hygiene				0.074
Adequate	61 (40.9)	1070 (40.0)	250 (23.4)	
Needs improvement	88 (59.1)	1608 (60.0)	329 (20.5)	
Sharing of water troughs with other animals				**0.025**
Yes	61 (40.9)	1136 (42.4)	222 (19.5)	
No	88 (59.1)	1542 (57.6)	357 (23.2)	
Use of equipment from other farms ^1^				**0.022**
Yes	59 (39.6)	1078 (40.3)	257 (23.8)	
No	90 (60.4)	1600 (59.7)	322 (20.1)	
Farmer				
Regular veterinary assistance				0.241
Yes	119 (79.9)	2128 (79.5)	450 (21.2)	
No	30 (20.1)	550 (20.5)	129 (23.5)	
Training in livestock production				**0.007**
Yes	25 (16.8)	441 (16.5)	74 (16.8)	
No	124 (83.2)	2237 (83.5)	505 (22.6)	
Secondary or higher education ^2^				**0.046**
Yes	24 (16.1)	448 (16.7)	81 (18.1)	
No	125 (83.9)	2230 (83.3)	498 (22.3)	

—, not applicable. Flock counts are reported only for variables measured at the flock level. ^1^ Includes the use of livestock handling tools and machinery shared between farms. ^2^ Yes: completion of secondary or higher education; No: education below secondary level. Variables shown in bold were selected for inclusion in the univariable mixed-effects logistic regression analysis based on statistical screening (*p* < 0.20) and/or biological relevance.

**Table 3:** Mixed-effects logistic regression analyses of factors associated with *T. gondii* seropositivity in small ruminants from northeastern Portugal.

Factor	Univariable Mixed-Effects OR (95% CI)	*p*-Value	Multivariable Mixed-Effects aOR (95% CI)	*p*-Value
Animal				
Species	1.93 (1.20–3.11)	**0.007**	1.74 (1.08–2.79)	**0.023**
(Sheep vs. Goats)				
Animal age	2.76 (2.18–3.48)	**<0.001**	2.78 (2.20–3.51)	**<0.001**
(Adult vs. Young)				
Breed	1.83 (1.24–2.70)	**0.003**	1.84 (1.24–2.72)	**0.003**
(Non-native vs. Native)				
Farm				
Production system (Semi-extensive vs. Intensive)	1.77 (0.40–7.77)	0.449	—	—
Permanent housing (No vs. Yes)	3.66 (1.22–11.02)	**0.021**	3.16 (1.04–9.60)	**0.042**
Presence of cats on the farm	1.19 (0.98–1.46)	**0.082**	1.69 (1.11–2.57)	**0.015**
(Yes vs. No)				
Contact with wildlife species	0.85 (0.56–1.31)	0.466	—	—
(Yes vs. No)				
Management practices				
Dedicated maternity area	1.28 (0.83–1.98)	**0.257**	—	— ^†^
(No vs. Yes)				
Water trough hygiene	0.82 (0.55–1.22)	0.322	—	—
(Needs improvement vs. Adequate)				
Sharing water troughs with other animals	0.79 (0.53–1.19)	**0.255**	—	— ^†^
(Yes vs. No)				
Use of equipment from other farms ^1^	1.34 (0.90–2.01)	**0.148**	—	— ^†^
(Yes vs. No)				
Farm hygiene	0.91 (0.61–1.36)	0.645	—	—
(Needs improvement vs. Adequate)				
Farmer				
Regular veterinary assistance	1.28 (0.79–2.08)	0.319	—	—
(No vs. Yes)				
Training in livestock production	1.49 (0.87–2.56)	**0.147**	—	— ^†^
(No vs. Yes)				
Secondary or higher education ^2^	1.27 (0.74–2.19)	0.382	—	—
(No vs. Yes)				

^1^ Includes the use of livestock handling tools and machinery shared between farms. ^2^ Yes: completion of secondary or higher education; No: education below secondary level. Abbreviations: OR, odds ratio; aOR, adjusted odds ratio; CI, confidence interval. Flock-level random-intercept variance = 0.948; intraclass correlation coefficient (ICC) = 0.224. Bold variables in the univariable column indicate variables considered for inclusion in the multivariable model. Bold estimates in the multivariable column indicate variables retained in the final model. Variables removed during backward elimination are indicated by ^†^.

## Data Availability

The original contributions presented in this study are included in the article. Further inquiries can be directed to the corresponding author.

## Abbreviations

The following abbreviations are used in this manuscript:

CI	Confidence interval
ELISA	Enzyme-linked immunosorbent assay
IgG	Immunoglobulin G
MAT	Modified agglutination test
OR	Odds ratio
aOR	Adjusted odds ratio
S/P%	Sample-to-positive percentage
